# A Single and Un-Adjuvanted Dose of a Chimpanzee Adenovirus-Vectored Vaccine against Chikungunya Virus Fully Protects Mice from Lethal Disease

**DOI:** 10.3390/pathogens8040231

**Published:** 2019-11-12

**Authors:** Rafael Kroon Campos, Lorena Preciado-Llanes, Sasha R. Azar, Cesar Lopez-Camacho, Arturo Reyes-Sandoval, Shannan L. Rossi

**Affiliations:** 1 Department of Microbiology and Immunology, University of Texas Medical Branch, Galveston, TX 77555, USA; rkkroonc@utmb.edu (R.K.C.); srazar@utmb.edu (S.R.A.); 2 The Jenner Institute, Nuffield Department of Medicine, University of Oxford, The Henry Wellcome Building for Molecular Physiology, Roosevelt Drive, Oxford OX3 7DQ, UK; lorena.preciado-llanes@ndm.ox.ac.uk (L.P.-L.); cesarlc@well.ox.ac.uk (C.L.-C.); 3 Department of Pathology, University of Texas Medical Branch, Galveston, TX 77555, USA

**Keywords:** vaccine, adenovirus-vectored, chimpanzee adenovirus, chikungunya virus, alphavirus, Togaviridae, joint swelling, 181/25, A129 mice

## Abstract

The mosquito-borne chikungunya virus (CHIKV) has become a major global health problem. Upon infection, chikungunya fever (CHIKF) can result in long-term joint pain and arthritis, and despite intense research, no licensed vaccine for CHIKV is available. We have developed two recombinant chimpanzee adenovirus-vectored vaccines (ChAdOx1) that induce swift and robust anti-CHIKV immune responses with a single dose, without the need for adjuvants or booster vaccines. Here, we report the vaccines’ protective efficacies against CHIKV infection in a lethal A129 mouse model. Our results indicate that a single, un-adjuvanted ChAdOx1 Chik or ChAdOx1 Chik ΔCap dose provided complete protection against a lethal virus challenge and prevented CHIKV-associated severe inflammation. These candidate vaccines supported survival equal to the attenuated 181/25 CHIKV reference vaccine but without the vaccine-related side effects, such as weight loss. Vaccination with either ChAdOx1 Chik or ChAdOx1 Chik ΔCap resulted in high titers of neutralizing antibodies that are associated with protection, indicating that the presence of the capsid within the vaccine construct may not be essential to afford protection under the conditions tested. We conclude that both replication-deficient ChAdOx1 Chik vaccines are safe even when used in A129 mice and afford complete protection from a lethal challenge.

## 1. Introduction

Chikungunya virus (CHIKV) is the etiological agent of chikungunya fever (CHIKF), an acute febrile illness that can result in long-term arthralgia, mainly in the distal joints of the extremities. Several, large-scale outbreaks have resulted in millions of infections worldwide [1]. Unlike many other arboviruses of clinical importance, the percentage of CHIKV infections resulting in symptomatic disease is high and the chronic phase of the illness can last for years [2]. Co-morbidities can exacerbate disease (reviewed in [3]). Furthermore, swelling and arthritis at the peripheral joints in the hands and feet results in a debilitating disease that directly affects the patient’s quality of life and places a burden on local caretakers and communities [2]. At present, there are no licensed treatments against CHIKF, and despite efforts made for over 50 years, no licensed vaccine for CHIKV is yet available (reviewed in [4,5]).

Adenoviruses have been used as viral vectors for decades. Recently, novel adenoviruses were prioritized, mainly due to pre-existing adenovirus immunity to common serotypes (e.g., Ad5) in the targeted human populations. Simian adenoviruses have been developed following concerns that pre-existing immunity to human adenoviral serotypes could limit their use as vaccines. Indeed, simian adenoviruses have minimal seroprevalence in humans [6]. The first report using a chimpanzee adenovirus as a viral-vectored vaccine was made in 2002 by Xiang et al. [7], who demonstrated the induction of immune responses to a rabies glycoprotein expressed by a chimpanzee adenovirus serotype 68. A novel vaccine vector was recently derived from the chimpanzee adenovirus isolate Y25 subgroup E. This viral vector known as ChAdOx1 was engineered at the University of Oxford as a replication-incompetent virus by deletion of its E1 region [8]. Recombinant ChAdOx1 vectors have successfully been used in vaccines against a wide variety of pathogens [9,10,11,12,13]. Furthermore, phase I clinical trials with ChAdOx viral vectors against diseases such as influenza (NCT01818362, NCT01623518), tuberculosis (NCT01829490, NCT03681860), MERS (NCT03399578), HIV (NCT03204617), malaria (NCT03203421), and meningitidis B (ISRCTN46336916) have demonstrated their safety and immunogenicity.

We have developed two ChAdOx1 vaccines that induce swift and robust anti-CHIKV immune responses upon a single dose, without the need for adjuvants or booster doses [14]. ChAdOx1 Chik (formerly known as ChAdOx1 sCHIKV) encodes a cassette expressing all CHIKV structural proteins (capsid, E3, E2, 6k, and E1), while ChAdOx1 Chik ΔCap (formerly known as ChAdOx1 sCHIKV ΔC) expresses all structural proteins but the capsid. In this manuscript, we report the ability of both replication-deficient chimpanzee adenovirus-vectored vaccines, ChAdOx1 Chik and ChAdOx1 Chik ΔCap, to induce immunity and afford protection in the highly susceptible and lethal A129 mouse model. Both vaccines fully protected against a lethal CHIKV challenge, viremia and weight loss. These data highlight the usefulness of the chimpanzee adenovirus vector platform as a CHIKV vaccine.

## 2. Results

### 2.1. ChAdOx1 Chik Vaccination is Well-Tolerated in Mice and Induces the Production of Neutralizing Antibodies

In order to test the efficacy of vaccines to prevent CHIKV-induced disease, the A129 mouse model was used. A129 mice were chosen for several reasons, including (1) their ability to determine whether a vaccine is safe, (2) a well-characterized and predictable course of disease, (3) a high susceptibility to lethal CHIKV infection in the absence of neutralizing antibodies, and (4) clear correlates of protection (i.e., reduction of viremia, decreased foot swelling and weight loss) needed to determine efficacy. The vaccines tested in this study include ChAdOx1 Chik and ChAdOx1 Chik ΔCap. The reference attenuated vaccine 181/25 was used as a positive control. Negative controls included an off-target vaccination (ChAdOx1 Zika, known as ChAdOx1 prME ΔTM) [13] and sham vaccination with PBS.

**Figure 1:**
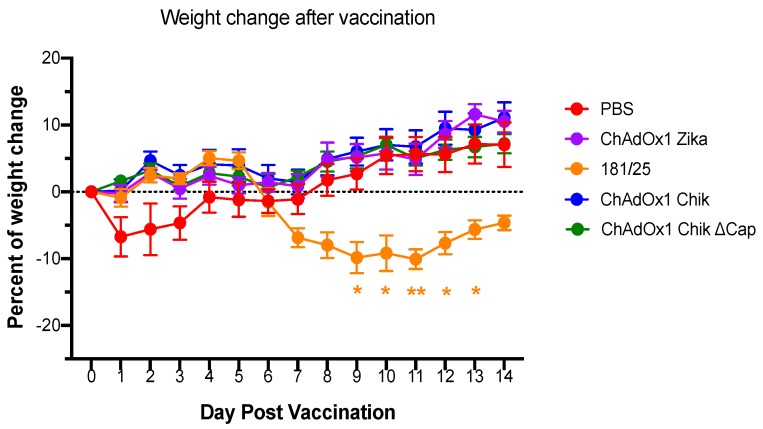
Vaccination with 181/25 results in weight loss, but this does not occur with any of the ChAdOx1 vaccines. Percentages of weight change following vaccinations are shown. The weights of A129 mice in each group were compared to their weights just before vaccination (day 0). Data are represented as means and SEMs. Two-way, repeated measures ANOVA with Dunnett’s (compared to PBS group); * *p* < 0.05; ** *p* < 0.01.

A129 mice tolerated intramuscular inoculation of most vaccines, as evidenced by the absence of weight loss during the 14 days following vaccination (Figure 1). The sudden weight loss during days 1–3 post in the PBS injected group corresponded to fighting observed in the cage and the resolution (separation) correlates to weight recovery in all mice. A significant weight loss was observed only in mice vaccinated with 181/25. These mice demonstrated signs of disease including a ruffled coat and weight loss between days 8 and 14 post vaccination. Mice continued to recover until the date of challenge. The comparison of the weight loss between PBS and 181/25-vaccinated mice was significant on days 8–12 post vaccination (Figure 1).

**Figure 2:**
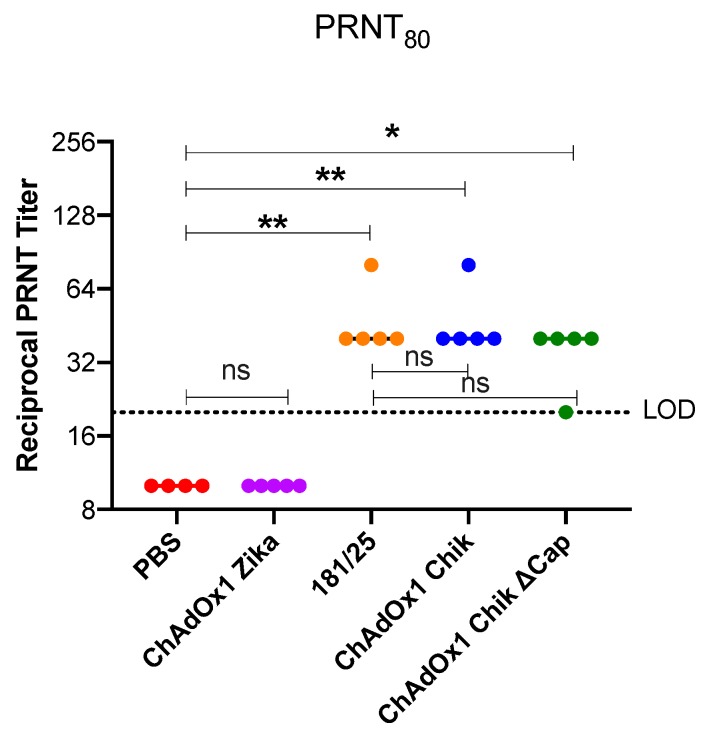
Reciprocal PRNT titers representing 80% neutralization (PRNT_80_). ChAdOx1 Chik and ChAdOx1 Chik ΔCap vaccines induce neutralizing antibody titers comparable to those induced by 181/25. PRNT titers on day 25 post vaccination. Horizontal dashed line represents the limit of detection (LOD) of the assay of 20. All values recorded as 10 had neutralization values < LOD. Dots represent titers for each animal; bars represent means and SEMs. One-way ANOVA with Sidak’s multiple comparisons test; * *p* < 0.05; ** *p* < 0.01.

Neutralizing antibodies are a key correlate of protection for alphavirus vaccines. Therefore, the plaque reduction neutralization test (PRNT) at the 50% and 80% cutoffs are important parameters of vaccine efficacy. All mice vaccinated with ChAdOx1 Chik, ChAdOx1 Chik ΔCap, or the 181/25 had measurable PRNT_50_ (Supplementary Figure S1) and PRNT_80_ titers at 3-weeks post vaccination (Figure 2). As expected, mice inoculated with PBS or with ChAdOx1 Zika had no neutralizing antibodies against CHIKV La Reunion strain (CHIKV-LR). There was a statistically significant difference between the PRNT_80_ titers elicited from ChAdOx1 Chik, ChAdOx1 Chik ΔCap, and 181/25-vaccination when compared to either PBS or ChAdOx1 Zika (one-way ANOVA with multiple comparisons, *p* < 0.0001).

### 2.2. ChAdOx1 Chik and ChAdOx1 Chik ΔCap Protect from Lethal CHIKV Challenge

**Figure 3:**
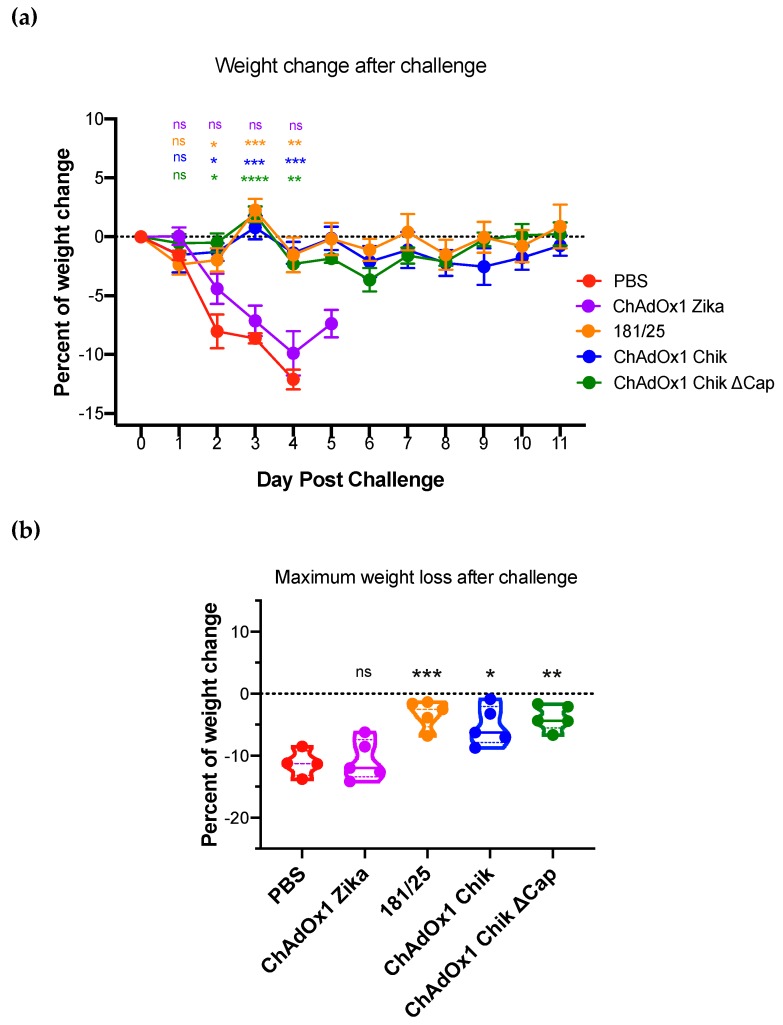
ChAdOx1 Chik and ChAdOx1 Chik ΔCap vaccines prevent weight loss of A129 mice following CHIKV-LR challenge. (**a**) The percentages of weight change after the challenge for each vaccine are shown. Data are represented as means and SEMs. Restricted maximum likelihood mixed model with Dunnett’s (compared to PBS group). * *p* < 0.05; ** *p* < 0.01; *** *p* < 0.001; **** *p* <0.0001. (**b**) Maximum weight loss recorded for each animal at any given timepoint after infection. Dots represent each mouse; data represented as violin plots with medians. One-way ANOVA with Dunnett’s (compared to PBS group); * *p* < 0.05; ** *p* < 0.01; *** *p* < 0.001.

One month after vaccination, mice were challenged with a lethal dose of CHIKV-LR (Backtiter: 9.7 × 10^4^ pfu/mouse) in the left rear foot. Weight loss and foot swelling are associated with CHIKV-caused disease in A129 mice; these parameters were measured daily for 11 consecutive days. The percentage of initial weight prior to challenge is shown in Figure 3a. Only mice from the PBS-treated and ChAdOx1 Zika-vaccinated groups lost weight after the challenge, which correlated with the low neutralizing protective antibodies by PRNT, all below the limit of assay detection. All mice vaccinated with ChAdOx1 Chik, ChAdOx1 Chik ΔCap, or 181/25 were fully protected from weight loss. Importantly, even the lowest weight recorded for each mouse along the whole post challenge period was significantly higher in ChAdOx1 Chik, ChAdOx1 Chik ΔCap, and 181/25 groups compared to PBS (Figure 3b).

One of the advantages of the A129 mouse model for studying CHIKV infection is the ability to measure survival following a challenge. In addition to protection from weight loss, all mice vaccinated with ChAdOx1 Chik and ChAdOx1 Chik ΔCap vaccines survived the challenge with CHIKV-LR (Table 1). These groups were significantly different from the PBS group, which failed to protect mice and resulted in morbidity, triggering euthanasia between days 4 and 5 post-challenge. The off-target ChAdOx1 Zika-vaccinated mice showed no statistically significant difference from PBS-treated mice (Table 1).

**Figure 4:**
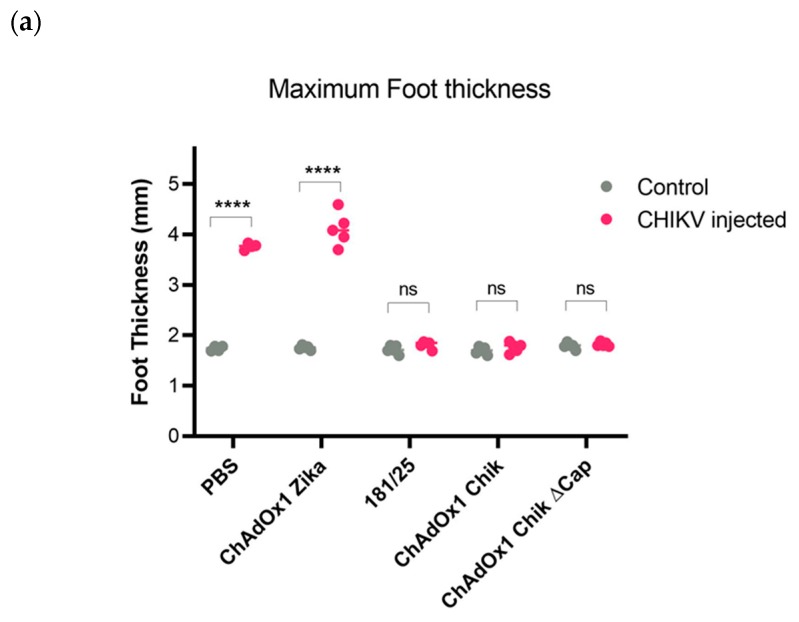

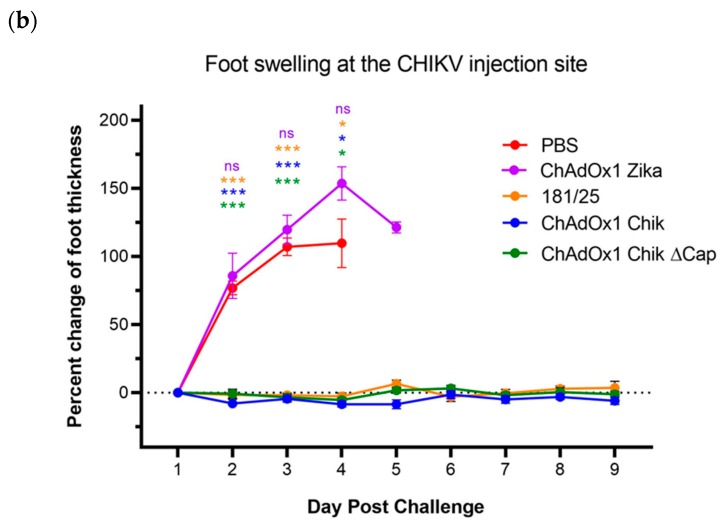
ChAdOx1 Chik and ChAdOx1 Chik ΔCap vaccines prevent foot swelling of A129 mice following CHIKV-LR challenge. (**a**) Maximum foot thickness in millimeters (mm) at any given timepoint following CHIKV infection. Comparison between the control foot (right foot) and the CHIKV injected foot (left foot). Dots represent each mouse. Multiple paired two-tailed *t*-tests with Holm-Sidak; **** *p* < 0.0001. (**b**) Percentages of CHIKV-induced foot swelling compared to baseline (day 1 post injection). Data represented as means and SEMs. Restricted maximum likelihood mixed model with Dunnett’s (compared to PBS group); * *p* < 0.05; *** *p* < 0.001.

Swelling is a hallmark of CHIKV infection in the injected foot. All mice that survived infection were also fully protected from swelling at the injection site. Conversely, all mice with weight loss and lethal disease had significant swelling starting on day 2 post challenge, which failed to resolve. Swelling was only seen in the CHIKV infected (left rear) foot and not the contralateral (right rear) control foot (Figure 4a). Swelling rapidly increased in PBS and ChAdOx1 Zika groups, peaking on day 4 post infection and failing to resolve before the animals reached the humane endpoint and were euthanized (Figure 4b). There was no significant swelling seen in ChAdOx1 Chik, ChAdOx1 Chik ΔCap, or 181/25-vaccinated mice, in either foot (Figure 4a,b).

**Figure 5:**
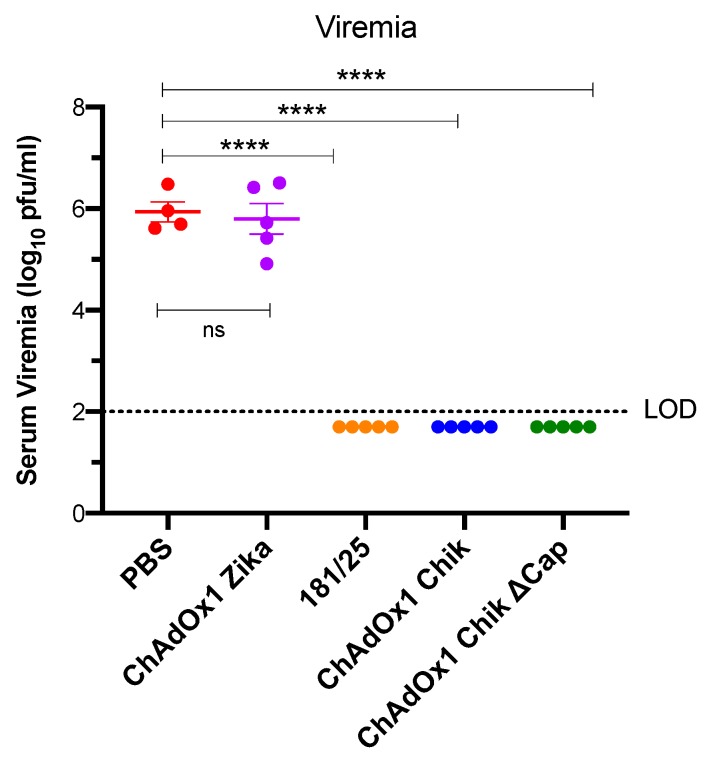
ChAdOx1 Chik and ChAdOx1 Chik ΔCap vaccines afford neutralizing immunity against the CHIKV-LR challenge in A129 mice. CHIKV viremia from serum collected on day 2 post challenge. Horizontal dashed line represents the limit of detection (LOD) of the assay at 2 log_10_ pfu/mL. Any value < LOD is recorded as 1.7 log_10_ pfu (1/2 LOD). Data are represented as means and SEMs; each dot represents a mouse. One-way ANOVA with Dunnett’s (compared to PBS group); **** *p* < 0.0001.

Previous studies have shown that viremia in A129 mice occurs on days 1–3 [15]. Therefore, to determine if the vaccine could protect against viremia, blood was taken from all mice on day 2 post challenge, which corresponds to the predicted day of peak viremia [15]. Consistent with survival and foot swelling, only the mice that were PBS-treated or ChAdOx1 Zika-vaccinated had a viremia of approximately 6 log_10_ pfu/mL (Figure 5). None of the other vaccinated mice had detectable viremia.

## 3. Discussion

Adenoviruses have been used as viral vectors for decades (reviewed in [16]). Human adenoviruses have been developed as CHIKV viral-vectored vaccines, and they have been shown to induce robust immune responses in mice [17,18]. These vaccines were tested in C57BL/6 mice, which produce high levels of neutralizing antibody against CHIKV. Foot infection of CHIKV in this mouse strain is not lethal and vaccines’ efficacy measurements include absence of viremia and protection from swelling. The present results are similar to the previous studies in that both ChAdOx1 Chik and ChAdOx1 Chik ΔCap vaccines trigger a protective immune response. The main correlate of protection against CHIKV-caused disease is likely to be neutralizing antibodies, based on a variety of studies [19,20]. All vaccinated mice in this study had measurable PRNT_80_ titers, with neutralizing antibodies above the ones necessary to neutralize CHIKV, albeit at lower levels than expected. This could be due to the mouse strain (C57BL/6 versus A129), level of immunocompetency (type I interferon competent versus deficient) and/or mouse age. The A129 mouse model is a sensitive model for CHIKV-caused disease; therefore, a lack of interferon type I response may negatively influence the production of neutralizing antibodies. Moreover, pairing this vaccine with appropriate adjuvants may improve the production of neutralizing antibodies and immunity against CHIKV. Human adenovirus vectored vaccines work well in mice with no pre-existing immunity to these adenoviruses. However, due to the high seroprevalence of the most popular human adenoviruses in the population [21,22], other primate adenoviruses with lower human seroprevalence like gorilla and chimpanzee ones have been pursued [6,23,24]. These primate adenoviruses have been shown here and by others to be effective vector platforms [9,10,11,12,23,25,26].

The strain of mouse chosen for vaccine studies for CHIKV disease is critical, since the measures of vaccine efficacy are strain dependent. Here, we use mice lacking the ability to respond to type I interferons because they are highly susceptible to lethal CHIKV infection [27]. A129 mice have been used successfully to test CHIKV vaccines since any vaccine that fails to produce a potent antibody response fails to protect against CHIKV challenge [28]. All ChAdOx1 CHIKV-vaccinated mice were protected in this model, even at a high challenge dose, highlighting the robustness of the single-dose vaccination. Furthermore, high titer viremias are produced from CHIKV infection in A129 mice [15], and ChAdOx1 CHIKV vaccines were able to reduce challenge viremia levels to undetectable levels on the day of highest anticipated titer. This feature is critical for arbovirus vaccines as it decreases the potential for mosquito transmission. Despite their usefulness in evaluating safety and efficacy, A129 mice also show mortality following CHIKV, which is not observed in CHIKV-infected patients. C57Bl/6 mice are often used for vaccine studies since they have a fully-intact type I interferon response, show foot swelling, and do not succumb to CHIKV infection. Given that the ChAdOx1 CHIK vaccines described here are safe and effective, further characterization, including dosing and long-term immunogenicity studies in C57Bl/6 mice is warranted.

The ChAdOx1 vectored vaccines described here were designed to express a multi-lineage mosaic protein with the aim to widen protection against all CHIKV lineages [14]. We hypothesized that as neutralizing antibodies against the CHIKV surface antigens correlate with protection, the capsid being the most internal structural protein might be less of a requirement to induce effective immunity. To test this, we designed an alternative vaccine with a CHIKV structural cassette lacking the capsid gene. There were no significant differences in survival, viremia, or weight loss between ChAdOx1 Chik- and ChAdOx1 Chik ΔCap-vaccinated mice following lethal CHIKV challenge under the conditions tested. Nevertheless, we are aware that differences in protection may emerge as conditions change, such as a reduction in vaccine dose or the assessment of long-term efficacy afforded in the presence or absence of the capsid as part of the vaccine.

The most commonly used strain to perform in vivo vaccine efficacy studies is the LR strain. However, multiple lineages of CHIKV are found worldwide and have slightly different phenotypes in the A129 mouse [15]. Regardless, all known CHIKVs are comprised of one serotype and antibody immunity against one lineage can be cross-neutralized by another [29]. Pre-challenge serum was tested by PRNT only against the LR strain here but future studies will expand upon this panel to confirm high-neutralizing antibody titers against all known lineages to add further evidence of the cross protective efficacy of the mosaic antigen evaluated.

CHIKF has become a major global health concern due to its ability to cause debilitating long-term joint pain and arthritis in infected individuals. Recent scientific efforts have resulted in promising CHIKV candidate vaccines, going from pre-clinical to phase I and II clinical trials; however, no vaccine has yet been licensed. There are multiple vaccines in development using a wide variety of approaches, including live-attenuated, protein subunit, viral-vectored, and nucleic acid-derived [4,30]. Each approach has its benefits and drawbacks. The chimpanzee adenovirus-vectored approach used here combines an enhanced safety profile on par with inactivated vaccines (as evidenced by no significant weight loss in A129 mice directly following vaccination) and immunogenicity comparable to live-attenuated vaccines (as shown by lack of viremia and swelling as well as survival of the lethal challenge). We believe this vaccine shows great promise and should be evaluated in nonhuman primates and clinical trials.

## 4. Materials and Methods

### 4.1. Cells, Viruses, and Vaccines Used

Vero CCL-81 cells (American Type Culture Collection, Manassas, VA, USA) were maintained in Dulbecco’s minimal essential media (DMEM, Gibco, Thermofisher Scientifc, Waltham, MA, USA) supplemented with 10% fetal bovine serum (FBS, Atlanta biologicals, Flowery Branch, GA, USA) and 1% penicillin/ampicillin (Gibco, Thermofisher Scientific, Waltham, MA, USA). Cell cultures were maintained in an incubator set to 37 °C with 5% CO_2_.

The viral-vectored vaccines constructed at the University of Oxford consisted of a chimpanzee adenoviral vector platform, ChAdOx1, which has a deleted E1 locus responsible for viral replication and the E3 gene [8]. The genetic cassette designed based on structural sequences of CHIKV was inserted into the Early gene (E1) that was deleted from the ChAdOx1 vector using an *attR1 attR2 Gateway*^®^ cassette (Invitrogen). The degree of conservation was analyzed and a consensus sequence from the three CHIKV lineages was utilized in the design of the synthetic gene. We constructed two vaccines, one with the whole structural cassette (ChAdOx1 Chik) and one without capsid (ChAdOx1 Chik ΔCap). ChAdOx1 Chik contains the full-length polyprotein of CHIKV, including capsid, 6k, and envelope (E) 3, E2, and E1. This plasmid was used as DNA template to further generate the E3, E2, 6K, E1 cassette with deletion of the capsid (ChAdOx1 Chik ΔCap) by PCR cloning. Transgenes were cloned into a pMono plasmid, under the control of a CMV promoter. More details have been described before [14]. ChAdOx1 Zika (Zika prME ΔTM) was used as a negative control [13]. The presence of the inserted cassettes into the ChAdOx1 vector was confirmed by PCR and sequencing. Virus were purified by cesium chloride (CsCl) gradient ultracentrifugation and assessed for sterility. The resulting products were titrated to obtain infectious units (IU) per mL and assayed by spectrophotometry to measure the number of virus particles (vp) per mL. ChAdOx1 Chik titer was 5.7 × 10^9^ IU/mL with a ratio of vp:IU of 29. ChAdOx1 Chik ΔCap titer was 1.8 × 10^9^ IU/mL with a ratio of vp:IU of 83. ChAdOx1 Zika titer was 1 × 10^10^ IU/mL with a ratio of vp:IU of 94. Vaccine excipient was made of 10mM Tris, 7.5% sucrose, 150nM NaCl, 0.1% Tween80 at pH 7.8. ChAdOx1 Chik, ChAdOx1 Chik ΔCap, and ChAdOx1 Zika were manufactured by the Viral Vector Core Facility at the Jenner Institute from the University of Oxford. CHIKV 181/25 vaccine was rescued from a cDNA clone as described in [31]. The 181/25 vaccine was developed by the United States Army Medical Research Institute of Infectious Diseases (USAMRIID) by passaging the Southeast Asian human isolate 15561 in MRC-5 cells 18 times [32]. Stocks were titrated from tissue culture media as listed above and frozen at −80 °C until ready to use. A cDNA clone encoding the LR strain of CHIKV was used to rescue the virus used in the challenge. Information regarding cDNA construction and rescue have been reported [33].

### 4.2. Animal Usage

A129 mice (type-I interferon receptor-deficient mice on the 129 genetic background) are maintained in sterilized caging in a breeding colony at UTMB. Mice of both genders were randomized into cohorts and vaccinated at 5-weeks-old. At this time, individual mice were ear-notch identified. All animal manipulations were done in accordance with an approved Institutional Animal Care and Use (IACUC) protocol (1708051). UTMB is an AALAS-approved facility. Any mouse reaching a humane endpoint were euthanized by CO_2_ asphyxiation, including paralysis, tremors, inability to move when stimulated, inability to eat/drink, or greater than 20% weight loss. Day of death was recorded as the day of euthanasia.

### 4.3. Vaccination

Immediately prior to vaccination, ChAdOx1 vaccines were thawed on ice and 181/25 was thawed at 37 °C, and then all vaccines were diluted in Dulbecco’s phosphate buffered saline (DPBS, Gibco, Thermofisher Scientific, Waltham, MA, USA) at room temperature and transported to the animal facility for vaccination. All vaccines except for 181/25 were shipped frozen in a ready-to-dilute formulation. ChAdOx1 vaccines were diluted to deliver a dose of 1 × 10^8^ IU per mouse and not back titrated after vaccination. The 181/25 vaccine was titrated directly after vaccination and determined to be 7.5 × 10^5^ pfu/mL (3.75 × 10^4^ pfu/mouse). All vaccinations were given intramuscularly in each hind leg (25 μL each leg) in an isoflurane-anesthetized mouse.

### 4.4. Challenge, Monitoring, and Foot Measurements

Thirty days post vaccination, mice were anesthetized with isoflurane and CHIKV-LR was injected intradermally into the left foot towards the ankle, on the portion which does not contact the ground, using a 28 G insulin syringe with a volume of 20 µL/dose. The right foot was not injected. The back titration of virus yielded a dose of 9.7 × 10^4^ pfu/mouse. Mice were weighed daily and all weights were compared to the weight on the day of challenge. Any mouse that lost more than 20% of their initial weight was humanely euthanized and death was recorded as the day of euthanasia. Swelling was measured as previously described [28]. The foot thickness was measured on both feet to ensure measurement consistency. Efforts were made to reduce the pain and stress of a foot injection, including limiting the challenge to only one foot and providing soft bedding and nesting material to the mice for the duration of the challenge phase. On day 2 post challenge, mice were anesthetized with isoflurane and blood was collected from the retro-orbital sinus with a capillary tube. Twenty-one days post challenge, all remaining mice were euthanized.

### 4.5. Blood Collection

On day 25 post vaccination and day 2 post challenge, mice were anesthetized with isoflurane and blood was collected retro-orbitally. Immediately after euthanasia, mice were exsanguinated by intracardiac bleeding. Blood was spun in microfuge tubes at 3380× *g* for 5 min, serum was removed and placed in a new tube, and samples were frozen at –80 °C.

### 4.6. Virus Titration

All virus and sera titrations were performed on Vero cell monolayers in 12-well plates as described previously [15]. Briefly, samples underwent 10-fold serial dilutions in media and were used to infect confluent Vero cell monolayers, after which a semisolid overlay containing 0.4% agarose was added and allowed to solidify. Plates were incubated at 37 °C with 5% CO_2_ for approximately 36 h before fixation with a 3.7% formaldehyde solution. Monolayers were stained with crystal violet to visualize plaques. Data are represented at pfu/mL with an assay limit of detection (LOD) of 100 pfu/mL. Any value less than the LOD was recorded as <LOD. For statistical and graphing purposes, values are assumed to be 50 pfu/mL (i.e., half of the LOD).

### 4.7. Plaque Neutralization Reduction Test

PRNT assays were performed against CHIKV-LR as previously described [29]. Sera were heat-inactivated at 56 °C for 1 h; then, diluted 2-fold in media after an initial 10-fold dilution. A known amount of virus was incubated with each serum dilution for 1 h, after which each virus-serum dilution was used to infect Vero cell monolayers in a 12-well plate. Plates were treated like a virus titration from that point on. The number of plaques in un-neutralized wells was 136, making a 50% reduction (PRNT_50_) yield 68 plaques and an 80% reduction (PRNT_80_) yield 28 plaques. The LOD was a titer of 1/20 and any titer below this was set at half of the LOD (titer of 1/10).

### 4.8. Statistical Analysis

Viremia data were transformed to log_10_ pfu/mL prior to analysis. Survival curve comparisons were made using Prism software and a log-ranked (Mantel–Cox) test. PRNT data were analyzed as reciprocal titers using a one-way ANOVA with Dunnett’s multiple comparison test. When the n number was sufficient, normality was assessed by D’Agostino and Pearson and by Kolmogorov–Smirnov tests. Multiple paired, two-tailed *t*-tests, and one-way or two-way ANOVA corrected by multiple comparison tests were used, as appropriate. Dunnett’s test was used when comparing to a control (PBS group) and Sidak’s when comparing selected pairs. In most cases data was represented by the mean of individual values and standard error of the mean (SEM). A significant difference was considered when the adjusted *p*-value was ≤ 0.05. Analyses were done using Prism v8.0 (GraphPad).

## Figures and Tables

**Table 1:** Survival of CHIKV-LR-challenged A129 mice.

Vaccine	# in cohort	Percent Survival	MDD ^1^	Significance ^2^
PBS	4	0% (0/4)	4.4 +/− 0.5	N/A
ChAdOx1 Zika	5	0% (0/5)	4 +/− 0	0.1763
181/25	5	100% (5/5)	N/A	0.0047
ChAdOx1 Chik	5	100% (5/5)	N/A	0.0047
ChAdOx1 Chik ΔCap	5	100% (5/5)	N/A	0.0047

^1^ Day of death ± standard deviation. ^2^ *p*-value, based upon log-ranked (Mantel–Cox) test compared to PBS.

## Supplementary Materials

The following figure is available online at https://www.mdpi.com/2076-0817/8/4/231/s1, Figure S1: ChAdOx1 Chik and ChAdOx1 Chik ΔCap vaccines induce neutralizing antibody titers comparable to those induced by 181/25 (PRNT_50_).
