# Supplementary material for: A Single and Un-Adjuvanted Dose of a Chimpanzee Adenovirus-Vectored Vaccine against Chikungunya Virus Fully Protects Mice from Lethal Disease
Source: Pathogens. 2019 Nov 12;8(4):231. doi: 10.3390/pathogens8040231 (PMC6963200; doi:10.3390/pathogens8040231)
Supplement: Supplementary file 1 [file pathogens-08-00231-s001.pdf]

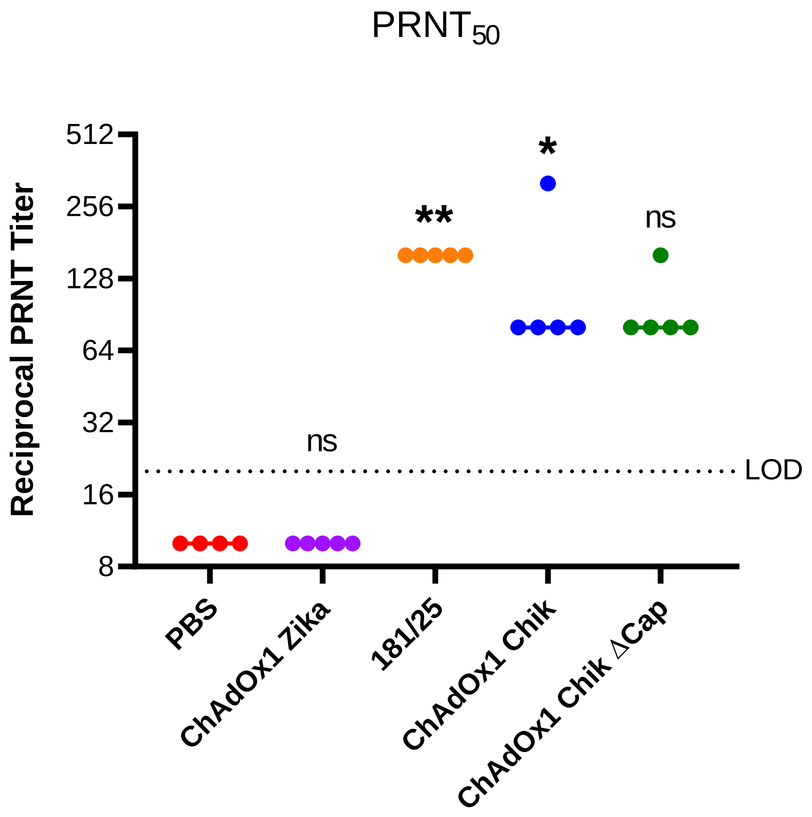

Figure 1. ChAdOx1 Chik and ChAdOx1 Chik ΔCap vaccines induce neutralizing antibody titers comparable to those induced by 181/25. PRNT titers on day 25 post vaccination. Horizontal dashed line represents the limit of detection (LOD) of the assay of 20. All values recorded as 10 had neutralization values <LOD. Reciprocal PRNT titers representing 50% neutralization ($PRNT_{50}$). Dots represent titers for each animal, bars represent mean and SEM. One-way ANOVA with Dunnett's (vs PBS); *$p < 0.05$, ** $p < 0.01$.
